# Nitroglycerin patch use in digital ischemia secondary to sepsis: a case report

**DOI:** 10.11604/pamj.2021.38.114.27279

**Published:** 2021-02-03

**Authors:** Devendrasing Vijaysing Jadhav, Derek Mendonca, Suresh Kotinatot, Shiva Shankar, Munira Al Mazmi

**Affiliations:** 1 Neonatal Intensive Care Unit, Al Jalila Children´s Specialty Hospital, Dubai, United Arab Emirates,; 2 Pediatric Plastic Surgery, Al Jalila Children´s Specialty Hospital, Dubai, United Arab Emirates

**Keywords:** Septicemia, ischemia, gangrene, nitroglycerin patch, case report

## Abstract

Sepsis results in intense disturbances in homoeostasis and is responsible for considerable morbidity and mortality in early infancy. Owing to insufficiency on part of infant to develop adequate inflammatory response to localize the infection, they usually progress to disseminated systemic infection, pneumonia and/or meningitis. We present the case of a 26 weeks preterm boy with acute digital ischemia in right index finger progressing to impending gangrene as a manifestation of septicemia. Use of topical nitroglycerin patch with meticulous monitoring successfully alleviated the impending peripheral gangrene without any adverse effects.

## Introduction

Bloodstream infection (BSI) is a common complication in neonates hospitalized for a long period in the neonatal intensive care unit (NICU) which is associated with high mortality and morbidity rates [1, 2]. Thromboembolism due to septicemic emboli can cause tissue ischemia progressing to gangrene of the involved region like digits if not detected promptly and treated early. The usual treatment of such ischemic injuries includes warming the contralateral extremity (reflex vasodilation) and immediate removal of any causative factor if found. Although incompletely evaluated anticoagulants such as unfractionated [3] or low molecular weight heparin [4] and thrombolysis with tissue plasminogen activator [3] are used with rapidly progressive ischemia, the use of Nitroglycerin (NGL) patch for peripheral tissue ischemia is rarely described in the literature which is mainly limited to vascular catheterization associated tissue ischemia. Here we report a unique case of successful use of NGL patch in neonatal intensive care unit for the treatment of peripheral tissue ischemia progressing to impending gangrene secondary to septicemia. Nitroglycerin patch used in our case did not show any adverse effects.

## Patient and observation

Baby boy with 26 weeks gestation and weight of 490 grams was born by caesarean section in view of pregnancy induced hypertension and absent Doppler signals. Mother´s serology was negative with no history of premature rupture of membranes. Baby required surfactant and medically treated for patent ductus arteriosus. Baby was referred to us at 11 weeks of postnatal age for spontaneous pneumoperitoneum. Closure of duodenal perforation and ileostomy were done after which baby remained stable. He was on room air and reached to full feeds within two weeks of surgery.

**Figure 1:**
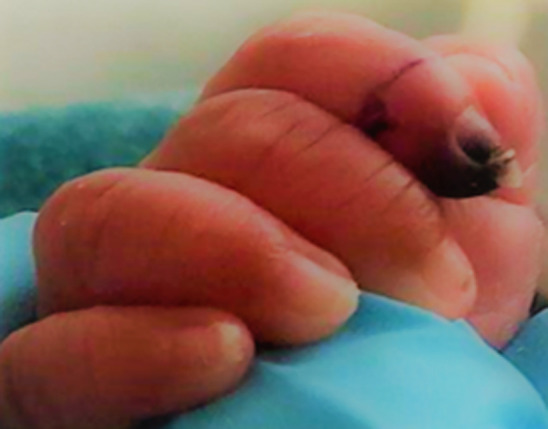
ischemia appeared on distal phalanx of right index finger; note the line of demarcation between the area with normal and abnormal perfusion

At eighteen weeks of postnatal age, he developed tachycardia, tachypnoea with fluctuations in SpO2. Seven hours into acute setback, fingers became cold with distal phalanx of index finger on right hand found to have dark bluish discoloration (Figure 1), however blood pressure was normal. Extremity was not pricked for any sampling, venous or arterial cannulation. Black streaky line over nail of left middle finger, index finger and thumb appeared within next 6-8 hours. List of investigations as shown in Table 1.

**Table 1:** investigations

Day 1*	WBC (total white blood cells)	2.12 x 10^9^/Litre
Day 1*	CRP (C reactive protein)	53 mg/L
Day 1*	Platelet count	32 x 10^9^/Litre
Day 1*	Blood culture (peripheral line)	Enterobacter cloacae (grew within 24 hours)
Day 1*	Blood gas (Methaemoglobin levels)	Normal
Day 1*	Coagulation work up	Normal
Day 1*	ECHO	Normal
Day 1*	Doppler study of both upper limbs till ulnar and radial arteries	Did not show any evidence of clot and blood flow through both arteries were adequate. However, we could not able to see beyond both these vessels because of obvious limitation of size of vessels
Day 2	Cerebrospinal fluid examination / culture	Normal
From Day 1 to Day 5 (during NGL patch use)	Blood gases with Methaemoglobin levels	Methaemoglobin levels remained less than 1.5
Repeated after 3 weeks	Follow up Cranial ultrasounds	Did not show any change from baseline

* When symptoms (Tachycardia, tachypnea and SpO2 fluctuations) started.

**Figure 2:**
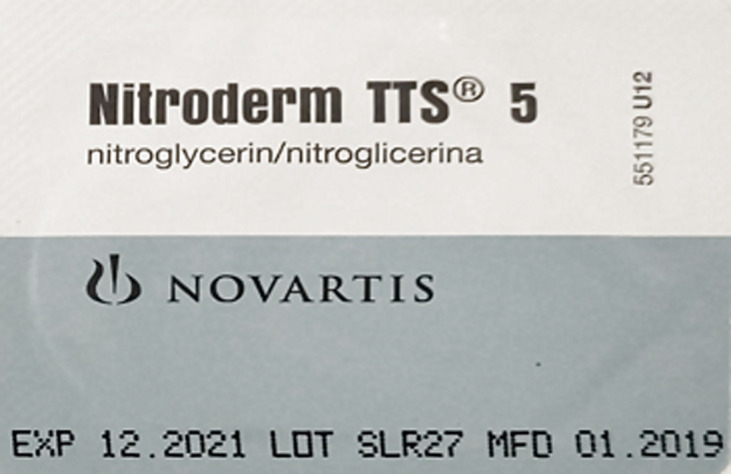
nitroglycerin patch used to treat ischemia of distal phalanx of right index finger

The diagnosis of peripheral tissue ischemia secondary to septic emboli was made. Antibiotics were started immediately after collecting blood sample to rule out sepsis in view of tachycardia, tachypnoea and SpO2 fluctuations. Contralateral limb warming was done to provide reflex vasodilatation. In consultation with plastic surgeon, within 24 hours of onset of ischemic changes, we started applying Nitroglycerin patch (Figure 2) 2.5 mg/kg (5 mg patch cut into two halves). It was applied twelve hours a day, extending up to 1 cm proximal to line of discoloration and continued for five days.

**Figure 3:**
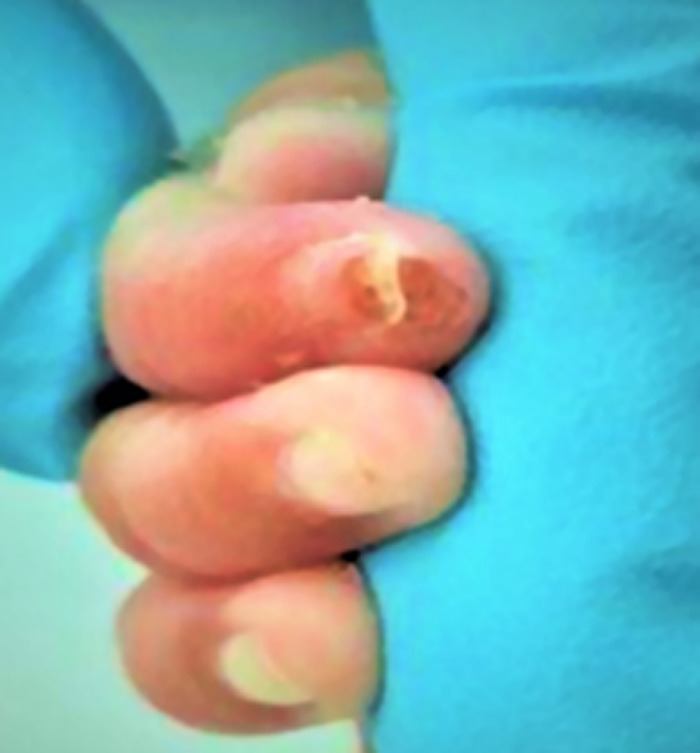
complete recovery of ischemic changes in distal phalanx after using; nitroglycerin patch; only superficial scab formation on fingertip is noted

After 24 hours of starting treatment, significant improvement in color and perfusion were noted locally. Further gradual improvement to complete recovery of digital ischemic changes were noted in 5 days with only scab formation on fingertip (Figure 3) and which completely healed in 2 weeks. Blood pressure and methaemoglobin levels remained stable throughout the course of NGL treatment. Cranial ultrasounds did not show any change from baseline. The time of events is listed in Table 2.

**Table 2:** timeline of events

Date	Event
28^th^ December 2019	Baby referred to our neonatal intensive care of spontaneous pneumoperitoneum.
28^th^ December 2019	Closure of duodenal perforation and colostomy.
26^th^ January 2020	Stable on room air with full oral tube feeds.
18^th^ February 2020	Symptoms Tachycardia, tachypnea, SpO2 fluctuations.
18^th^ February 2020	Sepsis screen sent, Antibiotics started: results revealed CRP 53 mg/L, Leukopenia, thrombocytopenia.
18^th^ February 2020	Right index fingers distal phalanx turned bluish black within 7 hours of symptoms (Image 1).
19^th^ February 2020	Blood culture grew Enterobacter cloacae (within 24 hours). Cerebrospinal fluid physical examination was normal.
	Nitroglycerin patch (Image 2) application started (within 24 hours).
20^th^ February 2020	Some improvement noted in color and perfusion of right index finger´s distal phalanx (after 24 hours of patch).
23^rd^ February 2020	Color and perfusion of right index finger´s distal phalanx become normal and so Nitroglycerin patch discontinued (Image 3). Methaemoglobin level remained normal.
In next 2 weeks after stopping Nitroglycerin patch	The scab formation on superficial skin of right index fingertip completely healed without any loss.
After 3 weeks from stopping Nitroglycerin patch	Brain ultrasound done showed no changes from baseline ultrasound findings.

## Discussion

Peripheral ischemic injuries are commonly described with wrong placement of catheter, intra-arterial catheters, peripheral vasospasm from vasoactive drugs or extravasation [5], however rare with sepsis. These can lead to complete gangrene and loss of the affected digits, if not treated timely. Platelets release cytokines, recruit leukocytes, interact with bacteria and endothelium which contribute to microthrombi formation [6]. These microthrombi may be responsible for deranged perfusion and gangrene of peripheries. The available therapeutic options are local infiltration of phentolamine or hyaluronidase which are of very limited benefit in the infant population, and their use demands either systemic administration or cutaneous injection [5]. This has led many of the intensive care unit practitioners to err on the side of caution and rely solely on conservative management. The use of NGL for neonatal peripheral ischemic injuries is described since 1988. Nitroglycerin is readily absorbed through intact skin and acts by relaxation of smooth muscle and dilatation of peripheral arteries and veins.

Akingbola O *et al*. [7] reported complete recovery of the injured tissue with the application of NGL except two infants, who probably experienced a prolonged tissue injury before its use. Dose of NGL therapy in infants ranges from 0.12 mg/kg to 2.5 mg/kg or 4 mm/kg (1.22 mg/kg) [8]. Gangrene process may be prevented in initial stages but once sets cannot be reversed completely; only further progress can be prevented. Hence starting early treatment on recognition is the key factor. Such a case with sepsis induced peripheral ischemic injury treated with topical nitroglycerin patch therapy with resolution of impending gangrene has not been previously reported. Nitroglycerin use is rarely associated with low blood pressure, high methaemoglobin level and intracranial hemorrhage [9].

High level of awareness of these potential side effects with close monitoring during the treatment period especially in the first 6 hours when the peak concentration occurs can help in preventing them from occurring. Recent study by Kim J *et al*. [10] on nitroglycerin patch showed that preterm infants in whom nitroglycerin patches were applied, 30 (83.3%) premature infants without necrosis improved without complications, 4 (11.1%) showed hypotension, and 2 (5.6%) showed skin damage. Our case report is unique as peripheral tissue ischemia and impending gangrene was developed as a part of sepsis, Nitroglycerin patch has been used successfully as opposed to NGL ointments in other reports, severe ischemia impending gangrene reversed after starting NGL patch therapy and saved the distal phalanx of right index finger and no side effects were noted.

## Conclusion

Septicemia was complicated by peripheral tissue ischemia which progressed to impending gangrene of right index finger even if blood pressure was normal. Nitroglycerin patch was applied for five days and during treatment gradually perfusion, color returned to normal. There were no side effects noted with the use of nitroglycerin patch. Nitroglycerin patch can play a role in the rescue therapy of digital ischemia. Considering the unpredictability of tissue injury events and the complexity in performing a systematic study, establishment of a registry may be paramount in forming future guidelines for the safe and successful use of topical nitroglycerin therapy in the infant population.
